# The Creative Brain Under Stress: Considerations for Performance in Extreme Environments

**DOI:** 10.3389/fpsyg.2020.585969

**Published:** 2020-10-30

**Authors:** Oshin Vartanian, Sidney Ann Saint, Nicole Herz, Peter Suedfeld

**Affiliations:** ^1^ Human Effectiveness Section, Toronto Research Centre, Defence Research and Development Canada, Toronto, ON, Canada; ^2^ Department of Psychology, University of Toronto, Toronto, ON, Canada; ^3^ Department of Psychology, University of Waterloo, Waterloo, ON, Canada; ^4^ Department of Psychology, University of British Columbia, Vancouver, BC, Canada

**Keywords:** creativity, stress, performance, environmental psychology, brain networks

## Abstract

Over the last 2 decades, we have begun to gain traction on the neural systems that support creative cognition. Specifically, a converging body of evidence from various domains has demonstrated that creativity arises from the interaction of two large-scale systems in the brain: Whereas the default network (DN) is involved in internally-oriented generation of novel concepts, the executive control network (ECN) exerts top-down control over that generative process to select task-appropriate output. In addition, the salience network (SN) regulates switching between those networks in the course of creative cognition. In contrast, we know much less about the workings of these large-scale systems in support of creativity under extreme conditions, although that is beginning to change. Specifically, there is growing evidence from systems neuroscience to demonstrate that the functioning and connectivity of DN, ECN, and SN are influenced by stress – findings that can be used to improve our understanding of the behavioral effects of stress on creativity. Toward that end, we review findings from the neuroscience of creativity, behavioral research on the impact of stress on creativity, and the systems-level view of the brain under stress to suggest ways in which creativity might be affected under extreme conditions. Although our focus is largely on acute stress, we also touch on the possible impact of chronic stress on creative cognition.

## Introduction

Human beings not only work under optimal conditions, but also under stressful conditions that require physical and psychological resilience for survival, performance, and growth (Suedfeld and Steel, 2000). There is indeed a large scientific literature on the impact of stress on psychological and physiological functioning, but it is only recently that this work has begun to focus on the impact of stress on large-scale networks in the brain, including their functional connectivity (Hermans et al., 2014; van Oort et al., 2017; see also Menon, 2011). The overarching aim of this manuscript is to review this nascent literature in an effort to improve our understanding of the impact of extreme environments – specifically those that cause stress – on creativity. This is made possible by virtue of the fact that the three large-scale networks in the brain that are impacted by stress are also precisely the ones that have begun to shape our understanding of the emergence of creative ideas in the neuroscience of creativity (Beaty et al., 2016). Toward that end, we will begin by reviewing our current understanding of the neuroscience of creativity, before moving to a discussion of the impact of stress on the functioning and connectivity of large-scale networks in the brain. In the process, we will selectively review the behavioral literature on the impact of stress on creativity. We hope that this exercise will improve our understanding of the behavioral effects of stress on creativity, by revealing the key neural systems and their interactions that could mediate that link.

## Neuroscience of Creativity: a Brief Historical Tour

Although electrophysiological studies of the neurological bases of creativity can be traced back to Martindale’s pioneering research five decades ago (e.g., Martindale and Hines, 1975), it was with the advent of modern neuroimaging techniques around the turn of the century that our understanding of the neuroscience of creativity has blossomed (for reviews, see Vartanian et al., 2013; Abraham, 2018; Jung and Vartanian, 2018). Much of the early exploratory work in this area was motivated by brain mapping approaches, and typically focused on discovering single, isolated brain regions that might underlie the generation of novel and useful thoughts. The tasks varied widely, including creative story generation, open-ended problem solving, drawing, divergent thinking, finding pragmatic links between incoherent sentences, and analogy and metaphor, to name a few. In addition, there was equal if not more variability in the neuroimaging methodologies used to study the brain, each of which was characterized by its own intricate analytic workflow, signal-to-noise ratio, and temporal and spatial resolution. As such, the early results were characterized by high levels of variability and inconsistency (for reviews, see Arden et al., 2010; Dietrich and Kanso, 2010).

Soon, however, a number of quantitative meta-analyses of this literature followed, which demonstrated an altogether different picture of the creative brain at work (Vartanian, 2012; Gonen-Yaacovi et al., 2013; Boccia et al., 2015; Wu et al., 2015; see also Cogdell-Brooke et al., 2020). These meta-analyses illustrated two points: First, there is no single brain region that drives creativity. Rather, the entire brain contributes to creative cognition. Second, and critically, the neural correlates of creativity are process-specific and domain-specific. For example, there are dissociable neural regions that contribute to creativity in the verbal vs. non-verbal (spatial) vs. musical domains (Gonen-Yaacovi et al., 2013; Boccia et al., 2015). Similarly, there are dissociable neural regions that contribute to processes related to creativity such as analogy vs. metaphor (Vartanian, 2012), as well as creativity tasks that involve generation vs. combination of ideas (Gonen-Yaacovi et al., 2013). As is the case with other higher-order constructs such as reasoning (Goel, 2007; Prado et al., 2011), this early body of work demonstrated that creativity is hierarchical and componential, and emerges from the flexible and dynamic reconfiguration of brain regions that contribute to its various instantiations. This picture is consistent with componential models of creativity (Amabile, 2012) and problem solving (Sternberg, 1980), according to which higher-order cognitive abilities are decomposable into specific sub-processes (e.g., semantic memory, attention, etc.). As such, brain regions that exhibit a degree of functional specificity in relation to those sub-processes contribute to the types of creativity that draw on those functions.

## Neuroscience of Creativity: From Regions to Networks

A significant shift in the neuroscience of creativity occurred when researchers began to focus on the contribution of large-scale networks rather than isolated brain regions to the emergence of creative thoughts. Those networks were initially discovered using the technique of resting-state connectivity, based on which one can identify brain regions that exhibit similar patterns of fMRI activity fluctuations, and can therefore be grouped into large-scale brain systems called “networks” (Zabelina and Andrews-Hanna, 2016). In other words, at any given time, regions within the same network (e.g., visual, language, somatomotor, etc.) are likely to exhibit correlated activity when the individual is engaged in a task or at rest (i.e., not engaged in a task). It is important to note that the seven large-scale networks that have been identified to date also exhibit differing patterns of between-network connectivity (Lee et al., 2012). These patterns of between-network connectivity can be conceptualized better when we consider that, despite their functional differences, some networks can work together to support the same type of cognition. For example, when the individual is engaged in externally-oriented cognition (i.e., responding to stimuli in the external world), the visual, somatomotor, and dorsal attention networks show high levels of between-network connectivity (Yeo et al., 2011; Buckner et al., 2013). This makes sense, given that in many circumstances such externally-oriented cognition requires one to attend to and process sensory input.

**Figure 1:**
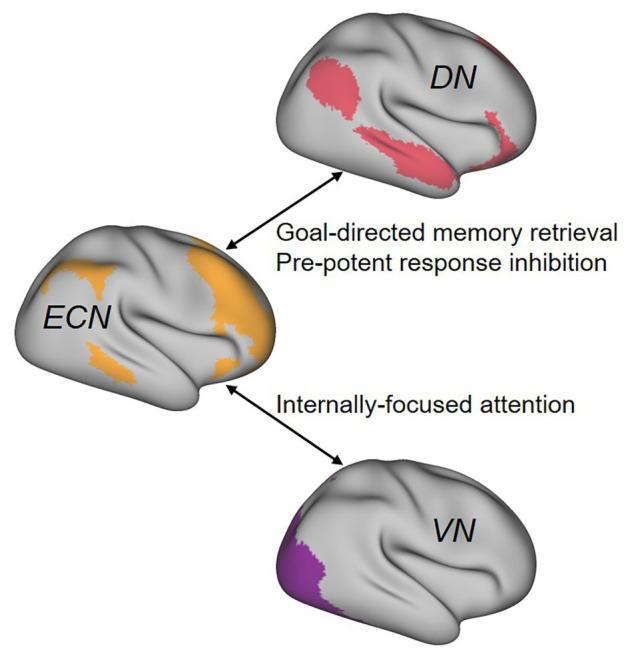
Cognitive mechanisms of brain network interactions during creative cognition. DN, default-mode network; ECN, executive control network; VN, visual network. Adapted with permission from Beaty et al. (2019).

Important for creativity researchers, a growing body of evidence has emerged to demonstrate that novel ideas emerge as a function of the dynamic interaction of the default network (DN) and the executive control network (ECN) in the brain (Beaty et al., 2016). Regions within DN are more active during task-unrelated thought than during task-related thought, and frequently come online during episodes of mind wandering, daydreaming, and imagination (Christoff et al., 2016; Raffaelli et al., 2020). In contrast, ECN is activated when the individual is engaged in tasks that require cognitive control. In most instances, DN and ECN activities are negatively correlated because individuals tend to be engaged in either task-related thought that necessitates cognitive control or task-unrelated thought that is not under top-down regulation. What is remarkable about creativity is that it represents a form of thinking that is supported by the dynamic interaction of these two modes of thought. Specifically, in the early phase of creative problem solving, when internally-oriented thoughts support idea generation, DN is relatively more active. In turn, in the later phases of creative problem solving, when the generated ideas are pruned to satisfy task demands, ECN is also engaged to exert top-down control to select appropriate output. Interestingly, aside from supporting goal-directed memory retrieval and inhibition of prepotent responses that represent some of its core functions, ECN may also facilitate internal orientation by shifting attention away from sensory input toward internally-generated thought processes carried out by DN (Benedek et al., 2016; Beaty et al., 2019; Figure 1).

An important early study that laid the groundwork for this interactive model was conducted by Ellamil et al. (2012), who presented design students with short verbal descriptions of the contents of books while in the fMRI scanner, and then instructed them to design book covers to represent them. In the generation phase, the participants drew and/or wrote down their ideas using an MRI-compatible tablet, whereas in the evaluation phase, they assessed the quality of their ideas and productions. During generation there was greater activation in DN, specifically the hippocampus. This is consistent with the constructive episodic simulation hypothesis, according to which memory and imagination involve flexible recombination of episodic details (e.g., people, places, and events; Schacter and Addis, 2007; Beaty, 2020). In other words, as we generate new ideas using imagination, it is likely that we mine our episodic memory to locate and flexibly recombine episodic details to support novel ideation. In turn, during evaluation not only was there activation in DN, but also additional activation in ECN, most notably in the dorsolateral prefrontal cortex that plays an important role in cognitive control. Additional analysis demonstrated that there was greater functional connectivity between DN and ECN during the evaluation phase, suggesting that there is close communication between those networks in the later stages of creative thinking when cognitive control is applied on the contents of generated ideas for their evaluation. Since then, data from several studies including musical improvisation (Pinho et al., 2016) and poetry composition (Liu et al., 2015) have also shown dynamic coupling between DN and ECN – interpreted to reflect the spontaneous generation of ideas derived from long-term memory and the evaluation of those ideas to meet specific task goals, respectively. Using dynamic causal modeling, Vartanian et al. (2018) have recently shown that ECN exerts unidirectional control over the activation of DN regions in the course of divergent thinking, supporting the causal model that underlies their interaction.

Beaty et al. (2015) used whole-brain functional connectivity analysis to highlight a network of brain regions associated with divergent thinking. This study was important because beyond DN and ECN, it also focused on the salience network (SN). SN has an important role to play in many types of higher-order cognition because it is involved in the detection and allocation of attention and neural resources to behaviorally relevant (i.e., salient) stimuli (Bressler and Menon, 2010; Menon and Uddin, 2010; Uddin, 2015). In this role, it can trigger the engagement of other networks based on their relevance to the task at hand. Analyses of Beaty et al. (2015) revealed that the posterior cingulate cortex (PCC) – a region that lies within the DN – exhibits increased functional coupling with ECN regions including the dorsolateral prefrontal cortex, as well as regions within SN such as the bilateral insula. Then, using dynamic functional connectivity analysis conducted in the course of engagement with the Alternate Uses Task, Beaty et al. (2015) demonstrated that the time-course of the coupling between the PCC and regions within SN and ECN varies as a function of the phase of the task. Specifically, the PCC showed early coupling with the insula and later coupling with the dorsolateral prefrontal cortex. There is evidence to show that one of the roles of SN is to facilitate switches between DN and ECN (Cocchi et al., 2013). As such, its involvement in divergent thinking could be to facilitate later coupling between DN and ECN.

Building on this work, Beaty et al. (2018) used connectome-based predictive modeling (CPM) – a machine learning algorithm for identifying functional connections in the brain that predict behavioral traits – to demonstrate that creative people are characterized by stronger functional connections between DN, ECN, and SN, and that this specific pattern of connectivity predicted their creativity scores. Interestingly, this dynamic interplay between DN and ECN has also been shown to be the case based on resting-state data, when people are not engaged in a task. Specifically, Beaty et al. (2014) reported that compared to less creative people, more creative people exhibit stronger DN-ECN coupling during rest, suggesting that at a fundamental neurological level more and less creative people may be distinguished by stable functional differences involving the coupling of key regions involved in creative cognition.

## Stress and Creativity: Behavioral Effects

It is generally assumed that stress has a detrimental effect on creativity. This assumption is not unreasonable: given that in the immediate aftermath of stress, physiological, and cognitive resources are reallocated to promote vigilance and survival (Hermans et al., 2014), it is likely that higher-order cognitive capacities that would otherwise support creative cognition would be shifted to meet those more urgent needs. Indeed, it has been demonstrated that stress has a negative impact on processes related to creativity, including task switching and cognitive flexibility (Steinhauser et al., 2007; Plessow et al., 2011, 2012). However, the impact of stress on creativity is not necessarily and universally negative, and depends in part on how stress-inducing the stressor is perceived to be, and the type of stress that is induced. For example, Byron et al.’s (2010) meta-analysis of 76 experimental studies that had examined the impact of stress on creativity demonstrated that uncontrollable stress leads to worse performance on creativity tasks, where uncontrollability was defined as the extent to which an individual believes that one’s actions can affect outcomes (Dickerson and Kemeny, 2004). In addition, they found that whereas high social-evaluative threats decreased creative performance, low social-evaluative contexts increased creative performance, where social-evaluative threats were considered to “occur when an aspect of self is or [can] be negatively judged by others” (Dickerson and Kemeny, 2004, p. 361). Thus, it appears that stress impacts creativity, but not necessarily in negative ways. Importantly, the findings are broadly consistent with appraisal models of stress (e.g., Lazarus and Folkman, 1984), according to which one’s perception of the stress and individual differences that underlie vulnerabilities to those stressors are important factors that influence the stress-creativity relationship.

## The Brain Under Stress: a Network View

**Figure 2:**
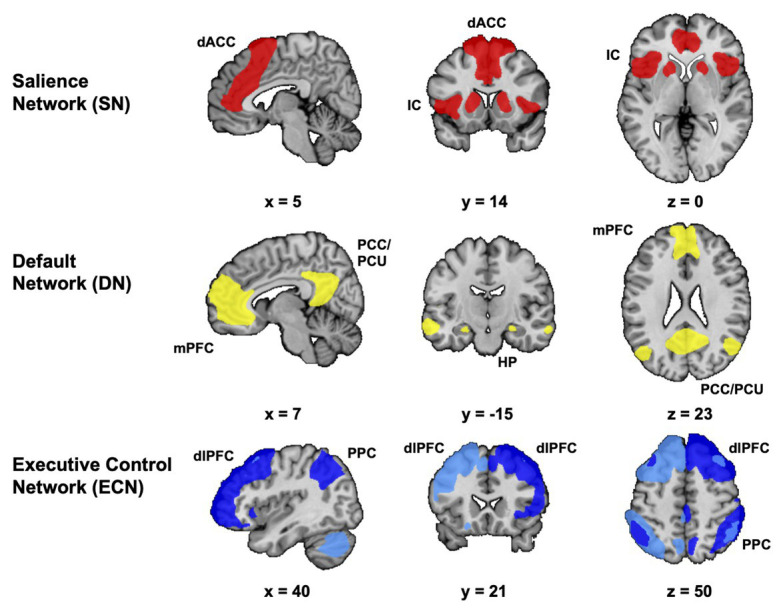
Major functional connectivity networks in the acute stress response. This figure is a schematic representation of the major functional connectivity networks relevant for the brain’s stress response. The core regions of the salience network (SN) are the insular cortex (IC), dorsal anterior cingulate cortex (dorsal ACC), temporal pole, and amygdala. The DN comprises the medial prefrontal cortex (mPFC), the posterior cingulate cortex/precuneus (PCC/PCu), and the inferior parietal lobule. The parahippocampal gyrus and hippocampus (HP) are strongly related to the DN. The ECN is centered on the dorsolateral prefrontal cortex (dlPFC) and posterior parietal cortex (PPC), and also includes part of the dorsomedial prefrontal cortex and frontal eyefields. Adapted with permission from van Oort et al. (2017).

At this point, we will consider how our knowledge of the workings of the brain can shed light on the impact of stress on creativity. Until recently this would have been difficult to do because with the exception of a few studies (e.g., Vartanian et al., 2014), we know very little about how the functioning of the creative brain is affected by various stressors. Fortuitously, however, we are now in a position to consider this question because a growing body of evidence from systems neuroscience has demonstrated that the three systems that support the emergence of creative thought under normal conditions are precisely the three systems whose functioning and connectivity is impacted by stress (for reviews, see Hermans et al., 2014; van Oort et al., 2017; see also Menon, 2011; Figure 2). As such, this offers one the opportunity to consider the ways in which the altered functioning and connectivity of SN, DN, and ECN can explain the impact of stress on creativity.

Following exposure to stress, a cascading series of physiological changes is triggered that ultimately impact neuronal function in temporally- and spatially-specific ways (Joëls, 2018). At the neuroendocrine level, central levels of catecholamines in the brain (e.g., norepinephrine and dopamine) increase rapidly and normalize shortly thereafter, whereas corticosteroid levels in the brain rise more slowly and remain high for a longer period of time (Hermans et al., 2014). The rapid rise in the level of catecholamines in the brain is associated with an increase in SN activity, and a decrease in ECN activity (Hermans et al., 2014; van Oort et al., 2017). Hermans et al. (2014) have argued that this represents a reallocation of resources to SN, a network that underlies orienting attention toward salient information in the environment (Menon, 2011). There is also a strengthening of the functional connectivity between SN and sensory cortices as the organism attends to sensory input (Li et al., 2014). Psychologically, this represents a hypervigilant state geared toward maximizing the likelihood of survival in the immediate aftermath of stress. This reallocation of resources comes at the cost of ECN, where activation diminishes or remains the same. Interestingly, and perhaps counterintuitively, there is an increase in DN activity immediately following exposure to stress. One reason might be that stress can lead to increased negative self-referential processing, which is known to engage the DN. Indeed, high social-evaluative threats are known to decrease creative performance (Byron et al., 2010). In addition, increased activity in the anterior sector of DN might be due to attempts to regulate emotion, another process that engages DN. Acute stress also brings about increased SN-DN functional connectivity, which may play an important role in memory consolidation given the association between SN and regions within DN that encode episodic memory such as the hippocampus (van Oort et al., 2017). After the stress has subsided, the allocation of resources to SN and ECN reverses, thereby restoring higher-order cognitive functions that are necessary for linking stressful events to the specific context, and to encode this information for future retrieval (Hermans et al., 2014; Joëls, 2018).

What does this mean for the creative brain under stress? Under normal circumstances, DN activity dominates in the early phase of creative problem solving. This is in stark contrast to what occurs in the acute response to stress where SN and sensory cortex activities increase (van Marle et al., 2010), as does their functional connectivity (Li et al., 2014). Furthermore, ECN activity decreases in the immediate aftermath of stress, and may not be prioritized in relation to SN activity until 1 h after the onset of stress (Hermans et al., 2014). Because creative cognition necessitates a dynamic interaction between DN and ECN, the downregulation of the latter will in all likelihood adversely impact the emergence of creative output (see Vartanian et al., 2018). In summary, despite the fact that DN activity increases in the immediate aftermath of stress, the reallocation of resources away from ECN to SN, as well as the increased functional connectivity between SN and sensory cortices for prioritizing attention to salient stimuli may well hamper the neural dynamics that support the emergence of creative thought.

## Acute Vs. Chronic Stress

In this paper, our focus has been on the impact of acute stress on the functioning of large-scale brain networks, with possible downstream impact on creative cognition. In other words, we have attempted to paint a picture of brain function in support of creative cognition in cases where a person encounters an extreme, stressful environment. However, quite aside from such acute forms of stress, one can also envision a host of chronic stressors that can negatively impact cognitive function, including creativity. Danese and McEwen (2012) reviewed the large literature on adverse childhood experiences, and demonstrated that such forms of chronic stress can have enduring impacts on the nervous, endocrine, and immune systems. Such long-lasting physiological changes (i.e., biological embedding) are perceived to represent the body’s allostatic response to chronic stress (Sterling, 1988). For example, adults with a history of childhood trauma exhibit smaller prefrontal cortex and hippocampal volume, with associated deficits in declarative memory. Given the important role that the semantic system is known to play in divergent thinking (see Beaty and Schacter, 2018; Kenett, 2018), it is plausible that such chronic forms of stress that have a deleterious impact on the nervous system may also negatively impact creative cognition.

Indeed, the functioning of the three large-scale networks that have been the focus of our discussion here are known to be affected by a wide host of psychiatric and neurological disorders that have long-lasting effects on brain structure and function. Review by Menon (2011) of the network neuroscience literature demonstrated that functional disruptions in the ECN as well as abnormalities in the intrinsic functional connectivity within the DN and SN are associated with virtually every major psychiatric and neurological disorder, including anxiety disorders, mood disorders, and schizophrenia, among others. Synthesizing this literature in his *Triple network model of psychopathology*, Menon (2011) argued that deficits in access, engagement, and disengagement of large-scale neural networks are a defining feature of psychopathology. To the extent that various psychiatric and neurological disorders can be viewed as chronic forms of stress, this body of research suggests a close correspondence between the neurological markers of acute and chronic stress at the network level, and suggests that a complete representation of the impact of stress on higher cognition including creativity requires an understanding of both its acute and chronic effects.

## Conclusion

Creative cognition has been shown to be supported by the dynamic interaction of DN, ECN, and SN. Furthermore, during divergent thinking, attention to sensory input is attenuated, and instead shifted to internally-generated thought. In contrast, in the acute response to stress (i.e., <1 h after the onset of stress) SN activity increases, whereas ECN activity decreases. There is also increased functional connectivity between SN and sensory cortices, as attention is directed to salient stimuli to maximize chances of survival. Although there is an increase in DN activity and DN-SN functional connectivity, this is likely related to self-referential cognition and emotion regulation rather than thought processes related to creativity. This pattern can help explain why under certain circumstances creativity is impacted negatively by stress, and points to network neuroscience as a useful avenue of research for studying the functioning of the creative brain under acute and chronic stress.

## Author Contributions

OV and PS wrote the manuscript. SS and NH conducted literature searches and edited the manuscript. All authors contributed to the article and approved the submitted version.

### Conflict of Interest

The authors declare that the research was conducted in the absence of any commercial or financial relationships that could be construed as a potential conflict of interest.
